# Possible internal viral shedding and interferon production after clinical recovery from COVID-19: Case report

**DOI:** 10.3389/fmed.2022.959196

**Published:** 2022-08-02

**Authors:** Asuka Ito, Takayuki Okada, Naoki Minato, Fumiyuki Hattori

**Affiliations:** ^1^Department of Anesthesiology, School of Medicine, Kansai Medical University, Osaka, Japan; ^2^Department of Cardiovascular Surgery, School of Medicine, Kansai Medical University, Osaka, Japan; ^3^Innovative Regenerative Medicine, Graduate School of Medicine, Kansai Medical University, Osaka, Japan

**Keywords:** SARS-CoV-2, COVID-19, post-COVID syndrome, long-COVID, type-I interferons

## Abstract

A 70-year-old man underwent off-pump coronary artery bypass grafting 28 days after his recovery from coronavirus disease 2019 (COVID-19), which was confirmed by a negative polymerase chain reaction (PCR) test result for severe acute respiratory syndrome coronavirus 2 (SARS-CoV-2) from a nasopharyngeal swab. The PCR test result was also negative for nasopharyngeal sampling 5 days prior to the surgery. However, his redundant saphenous vein and sputum through the endotracheal tube that was taken on the operative day showed the presence of SARS-CoV-2 by PCR. Immunohistochemical analysis of Spike and Nucleoprotein of the saphenous vein showed small clusters of each antigen-positive speckle. Ultrastructural imaging of the saphenous vein showed virus-like particles. The cell-based assay suggested that the patient’s serum contained a higher concentration of type-I interferons than that of healthy control sera. These observations suggest that internal viral shedding and, to some extent, innate immune responses continue after COVID-19 recovery.

## Introduction

The novel coronavirus, severe acute respiratory syndrome coronavirus 2 (SARS-CoV-2), has spread worldwide and infected at least 490 million people in April 2022. Coronavirus disease 2019 (COVID-19) has several pathological features, including delayed immune response (1) and post-COVID (long-COVID) syndrome (2). SARS-CoV-2 is believed to have been transferred from bats to humans. In bats, coronaviruses do not cause any immunoresponses (remain asymptomatic), suggesting that they are tolerated, that is, the viruses can escape the audit of intracellular innate immunity (3). It may not be a misunderstanding that at least part of this ability may work in humans as well. Actually, post-mortem histological analyses revealed a wide spectrum of tropism of SARS-CoV-2 and its long-term existence in human organs (4).

Longer asymptomatic periods accompanied by viral shedding after infections are a characteristic of SARS-CoV-2 infection. These periods are caused by the broad and efficient disruption of the host intracellular innate immune system by SARS-CoV-2, including the suppression of viral antigen presentation by MHC Class I (5). Therefore, infected cells cannot inhibit viral shedding or induce their own removal by killer T-cells. These defense systems against viral infection are normally mediated by the secretion of type-I interferons (IFNs). However, it has been suggested that type I IFN-mediated cellular immune responses are suppressed during the early phase of SARS-CoV-2 infection (1). In contrast, strong type I IFN-mediated immune responses are suggested to contribute to patient deterioration in the late phase of severe cases (6). However, to the best of our knowledge, no report regarding type I IFNs in clinically recovered patients has been published thus far.

Interferon therapy causes several side effects (7, 8). We noticed that IFN administration and post-COVID syndrome cause similar symptoms (2), including fatigue, exhaustion, weakness, dizziness, hair loss, loss of appetite, heart palpitations, depression, anxiety, high temperature, joint pain, diarrhea, stomachache, cough, headaches, sore throat, and skin rashes.

Here, we report a case in which the patient had clinically recovered from COVID-19; however, the virus continued to replicate internally, and the patient had relatively higher levels of type-I IFN in the serum. This report might be valuable for understanding the characteristics of post-COVID syndrome.

## Case description

**FIGURE 1:**
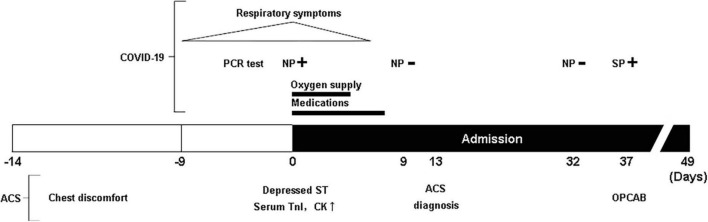
A schematic view of the patient’s clinical history. NP, nasopharyngeal; SP, sputum through intubation tube; TnI, Troponin I; CK, creatine phosphokinase-MB isozyme.

A 70-year-old man presented to our emergency hospital with dry cough and shortness of breath. He experienced gradual worsening of his symptoms for 10 days since their initiation. On physical examination, the patient’s oxygen saturation was 95%. Computed tomography (CT) of the chest revealed bilateral and peripheral ground-glass opacities. The polymerase chain reaction (PCR) for SARS-CoV-2 sampling from his nasopharyngeal swab was positive. He was admitted to the intensive care unit for COVID-19 and was administered oxygen, methylprednisolone, remdesivir, and tocilizumab for 4 days, 5 days, 3 days, and 1 day, respectively, which improved his dyspnea and chest CT findings (Figure 1).

His medical history included diabetes mellitus and hypertension. Approximately 1 month before admission, he had experienced paroxysmal chest pain. It was transient; therefore, he did not go to the hospital. On the day of admission, an electrocardiogram demonstrated an inverted T wave in V1-3 and non-specific ST-segment depression. Serum levels of troponin I and creatine kinase MB were elevated, suggesting acute coronary syndrome (ACS). These levels decreased in 1 day. Cardiac echocardiography revealed no wall motion abnormalities and a normal ejection fraction. Although the patient complained of no chest symptoms after admission, ACS was suspected. Therefore, the patient was administered nitrates with continuous treatment for his respiratory symptoms, waiting for further cardiac investigation. The PCR test result of his nasopharyngeal swab 9 days after admission was negative. He underwent coronary angiography 14 days after admission. Coronary angiogram demonstrated three-vessel disease of the left main trunk, which required coronary artery bypass grafting (CABG) (Figure 1).

Coronary artery bypass grafting was scheduled using an off-pump procedure, 37 days after admission. The patient was confirmed to have a negative PCR test result with his nasopharyngeal swab 5 days before off-pump coronary artery bypass grafting (OPCAB). One hour before entering the operating room, intra-aortic balloon pump (IABP) was inserted through the femoral artery under local anesthesia. Residual blood at the time of IABP insertion was preserved for analysis. During the OPCAB procedure, the saphenous vein was harvested for grafting, and residual tissues after anastomosis were preserved for analysis. Surgery was performed uneventfully. He was transferred to the postoperative intensive care unit, intubated, and sedated. Suctioning from the endotracheal tube was performed to remove the secretions during intubation. The secretion was used for PCR testing for SARS-CoV-2. The result of the PCR test was positive.

The patient was discharged on the 12th day following the surgery without any complications (Figure 1). At present, the patient is in good physical condition and did not present with cardiovascular complications as well as post-COVID-19 syndrome, 18 months after OPCAB.

### RNA extraction and laboratory real-time-polymerase chain reaction assay

**FIGURE 2:**
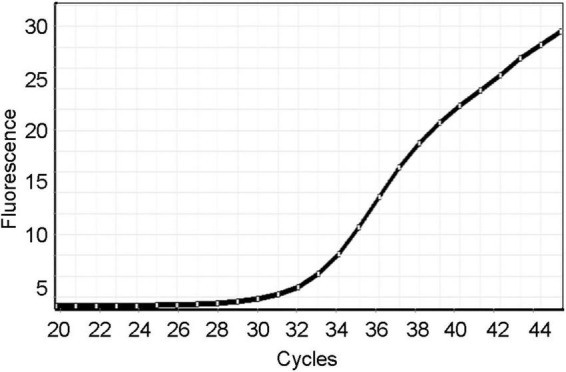
The amplification plot of the laboratory real-time RT-PCR using a nucleocapsid phosphoprotein gene-specific primer set.

Total RNA was extracted from the saphenous vein using Isogen^®^ (Nippon Gene, Japan), according to the manufacturer’s instructions. Reverse transcription of one microgram of RNA was performed using RiverTraAce^®^ (Toyobo, Japan) in the presence of random hexamers and oligo(dT). To investigate virus replication, quantitative PCR was performed using the Power SYBR Green RT-PCR master mix (Applied bioystems, United States) in the presence of a nucleocapsid phosphoprotein gene-specific primer set designed by the National Institute of Infectious Diseases, Japan. The primer sequences synthesized in the 5′-to-3′ direction were CACATTGGCACCCGCAATC as the forward primer (N_Sarbeco_F1) and GAGGAACGAGAAGAGGCTTG as the reverse primer (N_Sarbeco_R1). The amplification plot is shown in Figure 2.

### Immunohistochemistry

**FIGURE 3:**
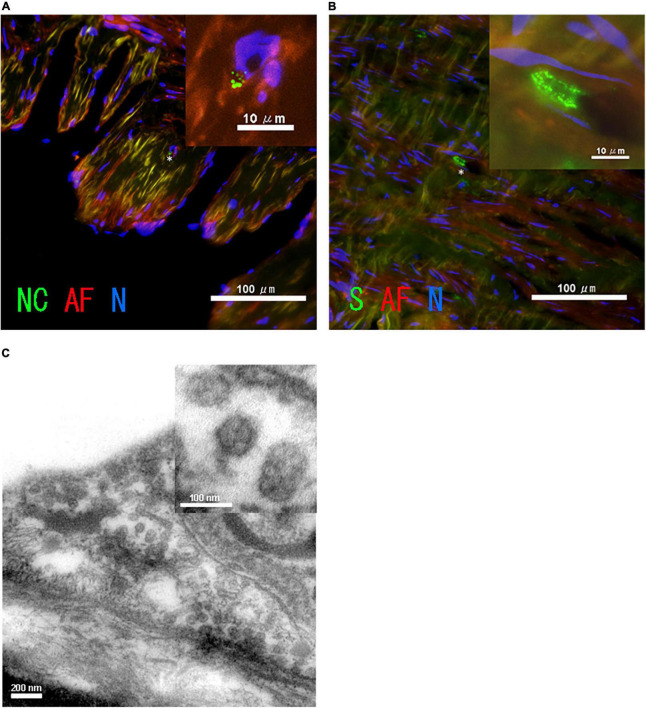
Images of SARS-CoV-2 in the patient’s saphenous vein. Immunohistochemistry of the saphenous vein of **(A)** SARS-CoV-2 nucleocapsid, NC (green), and **(B)** spike, S (green). The asterisk in A indicates the position of the enlarged image shown in the inset. Autofluorescence, AF (red), and nuclear staining with DAPI, N (blue), are also shown. **(C)** Image of transmission electron microscopy of saphenous vein.

Redundant tissue from the saphenous vein was fixed with 4% *para*-formaldehyde (pH 7.0). The fixed tissue was soaked in 30% sucrose (wt/vol) in Tris-buffered saline containing 0.1% tween-20 (TBS-T) and cryosectioned. Tissue sections were incubated with a primary antibody, a 1:100 diluted mouse anti-spike antibody (GeneTex, Irvine, CA, United States 6H3) or a 1:100 diluted mouse anti-nucleocapsid antibody (GeneTex, Irvine, CA, United States 1A9) at 4°C overnight. The sections were washed with TBS-T four times prior to being incubated with the secondary antibody, 1:200 diluted donkey Alexa Fluor 488 anti-mouse IgG (H + L) (Thermo Fisher Scientific, Waltham, MA, United States A21202), at room temperature for 30 min. After nuclear staining with DAPI (Thermo Fisher Scientific, Waltham, MA, United States) was performed, fluorescence signals were observed using fluorescence microscopy (Eclipse Ti2; NIKON, Tokyo, Japan). Immunohistochemistry revealed small clusters of antigen-positive speckles distinguished by autofluorescence (Figures 3A,B).

### Transmission electron microscopy assay

The saphenous vein was fixed with the Karnovsky’s formulation of 2% *para*-formaldehyde and 2.5% glutaraldehyde 1-mM CaCl_2_ in a 0.1-M HEPES buffer and then post-fixed with 1.0% osmium tetroxide in a 0.1-M HEPES buffer and 0.1-M sucrose. After additional buffer washes, the samples were dehydrated with a graded ethanol series and embedded in epoxy resin. Thin sections were cut using a Leica ultramicrotome, stained with 1% uranyl acetate for 30 min, and washed with distilled H_2_O. A Joel transmission electron microscope (JEM-1400A) was used for observation at 120 kV. Digital images were acquired using a Bioscan Camera Model 792 (Gatan, Inc., Japan) controlled by a digital micrograph 3.4 (Gatan, Inc.). Viral particles were observed in the saphenous vein (Figure 3C). An area devoid of viral particles was also observed (Supplementary Figure 1).

### Cell-based type-I interferon assay

**FIGURE 4:**
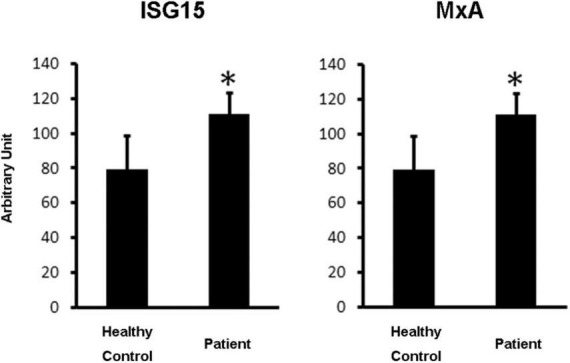
A cell-based type-I IFN assay. HepG2 cells were stimulated with the patient’s serum and healthy control serum at 10% concentration for 18 h (*N* = 3). The cDNA was amplified separately. Error bars indicate standard deviations. Statistical significance (*p* < 0.05) is indicated by asterisks.

HepG2 cells were cultured in a 12 well plate. Cells that reached 60% confluence were stimulated with patient serum and healthy control serum from a volunteer with no history of SARS-CoV-2 infection at 10% concentration for 18 h. We performed three independent experiments. Total RNA was recovered from each well by Isogen (Nippon Gene, Japan). Of the total RNA, 500 ng of RNA was reverse-transcribed using the Versa cDNA Synthesis Kit (Thermo Fisher Scientific, Waltham, MA, United States). One microliter of cDNA was amplified with primers (human ISG15 Fw: CACCTGAAGCAGCAAGTGAGCGGGCTGGAG, human ISG15 Rv: CCGCAGGCGCAGATTCATGAACACGGTGCT, human MxAFw: GCCAGCAGCTTCAGAAGGCCATGCTGC AGC, human MxARv: GGGCAAGCCGGCGCCGAGCCTGCG TCAGCC, human 18S Fw: TCAACTTTCGATGGTAGTC GCCGT, human 18S Rv: TCCTTGGATGTGGTAGCCGTTTCT) using the Power Cyber Green master mix (Thermo Fisher Scientific, Waltham, MA, United States) and quantitative PCR system, Rotor gene 2 (QIAGEN, Netherlands). Serial dilutions of one of the samples were amplified using each primer pair for quantification. The quantified expression levels were normalized to the result of 18S ribosomal RNA that was used as the internal control. The data obtained from three independent wells are expressed as mean ± standard deviation. Statistical analyses were performed using the Microsoft Excel Student’s *T*-test. Statistical significance was set at *p* < 0.05. As shown in Figure 4, type-I IFN stimulated genes (9), interferon-stimulated gene 15 (ISG 15) (10) and myxovirus resistance A (MxA) (11) expression levels were significantly higher in the HepG2 cells stimulated by the patient’s serum.

## Discussion

After the results of the first routine PCR test with nasopharyngeal swabs for SARS-CoV-2 were confirmed to be negative, the patient demonstrated no noticeable symptoms related to COVID-19. The patient was charged in COVID-19-free hospital until he underwent a sputum PCR test, suggesting the absence of *de novo* infection with SARS-CoV-2. Real-time PCR testing for SARS-CoV-2 performed in our laboratory using a surplus of tissue and bronchial sputum in the patient revealed possible internal, but not external, shedding of SARS-CoV-2 particles. In particular, the SARS-CoV-2 particle-like features in the immunohistochemical and ultrastructural images of the saphenous vein were strongly indicative of internal viral shedding.

It has been previously suggested that sputum PCR testing can provide clinically significant indications for persistent viral shedding (12). An increasing number of studies suggest that persistent viral shedding occurs in the lower part of the airways (13) and gastrointestinal tract (14), even after nasopharyngeal swabs demonstrate negative results. In this report, we propose the application of a PCR test to sputum samples obtained during airway intubation.

Here, we suggest the possibility of persistent viral shedding in the internal circulatory system. Although several postmortem studies of severe cases have been conducted (4), no studies have been conducted that investigate the persistence of SARS-CoV-2 particles in people who have recovered from non-severe COVID-19. Larger studies are needed to obtain sufficient information on the number of recovered patients who sustain internal viral shedding.

Persistent viral shedding may induce low-level secretion of type I IFNs; however, this may not lead to effective elimination of the infected cells. This report is the first to provide evidence of persistent type I IFN production in a patient after clinically recovering from COVID-19. Further research investigating how SARS-CoV-2 can escape detection by the immune system for prolonged periods under the partial activation of innate immunity is necessary.

We noticed a similarity between post-COVID symptoms (2) and the side effects of type I IFNs (8) and presented a possible case of this. In this report, we presented a novel theory regarding the hypothetical relationship between post-COVID-19 symptoms and the persistently low production of type I IFNs in the circulatory system.

## Data availability statement

The original contributions presented in the study are included in the article/Supplementary material, further inquiries can be directed to the corresponding author.

## Ethics statement

The studies involving human participants were reviewed and approved by the Kansai Medical University. The patients/participants provided their written informed consent to participate in this study.

## Author contributions

AI, TO, NM, and FH developed the concept and design of the study. TO and NM performed the surgery and collected the surplus samples. AI and FH performed the laboratory work to obtain the data. All authors contributed to the article and approved the submitted version.

## Supplementary material

Supplementary Figure 1 Reference image of transmission electron microscopy of saphenous vein. The area shown is near Figure 3C, but there are no viral particles.
