# Supplementary figures and images for: Possible internal viral shedding and interferon production after clinical recovery from COVID-19: Case report
Source: Front Med (Lausanne). 2022 Aug 2;9:959196. doi: 10.3389/fmed.2022.959196 (PMC9379344; doi:10.3389/fmed.2022.959196)

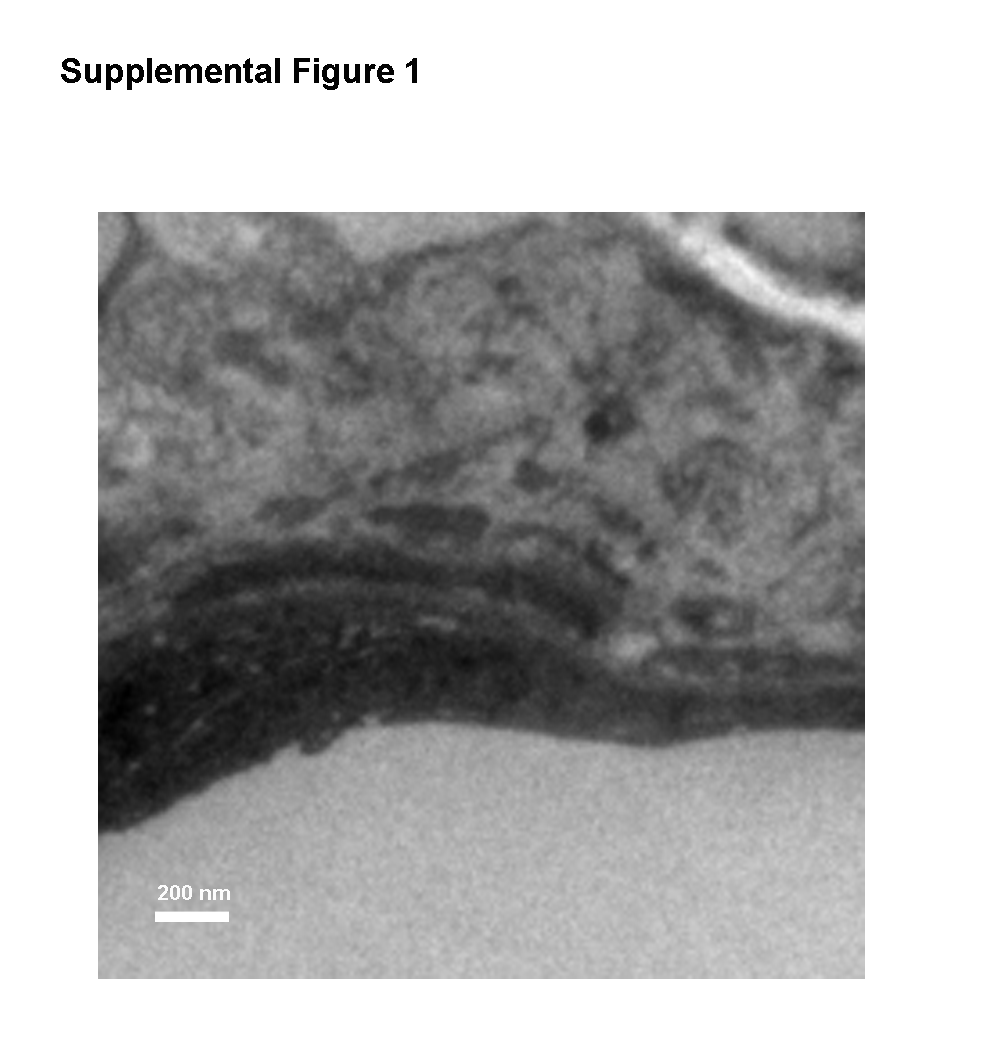

Supplement: Supplementary Figure 1 — Reference image of transmission electron microscopy of saphenous vein. The area shown is near Figure 3C, but there are no viral particles. [file Image_1.TIF]
